# Research trends on human trafficking: a bibliometric analysis using Scopus database

**DOI:** 10.1186/s12992-018-0427-9

**Published:** 2018-11-08

**Authors:** Waleed M. Sweileh

**Affiliations:** Department of Physiology, Pharmacology/Toxicology, Division of Biomedical Sciences, College of Medicine and Health Sciences, An-Najah National University, Nablus, Palestine

**Keywords:** Human trafficking, Bibliometrics, Research analysis, Global health

## Abstract

**Background:**

Human trafficking is a crime against humanity. It is also a serious threat to global health and security. Globalization has made human trafficking an easier task for the criminal organizations. No data are available on the volume, research trends, and key players in this field. Therefore, the aim of this study was to assess the research activity and research trends on human trafficking.

**Methods:**

A bibliometric method was adopted. Literature published in academic journals indexed in Scopus database was retrieved. The study period was set from 2000 to 2017.

**Results:**

Two thousand forty-four documents were retrieved. The average number of authors per document was 1.9. Over one third (*n* = 771; 37.7%) of the retrieved documents were about sex trafficking, 616 (30.1%) were about labor trafficking/forced labor, 199 (9.7%) were about child trafficking, and 138 (6.8%) were about organ trafficking. One third (*n* = 707; 34.6%) of the documents were in health-related fields while 1526 (74.7%) were in social sciences and humanities. The USA ranked first (*n* = 735; 36.0%) regarding the number of published documents. Geographic distribution of the retrieved document showed that world regions with a high prevalence of human trafficking had the least research contribution. International research collaboration has a limited contribution to the retrieved literature. The *Harvard University* (USA) was the most active institution (*n* = 39; 1.9%). *International Migration* (*n* = 35; 1.7%) was the most active journal in publishing documents on HT. Documents published in *Transplantation* journal received the highest number of citations per document (25.5) and two of the most cited documents were about organ trafficking.

**Conclusion:**

There was an under-representation of health-related literature on human trafficking. Literature on sex trafficking dominated the field of human trafficking. Research networks and research collaboration between the source and destination countries is important. Future research plans need to focus on health issues and on exploited/trafficked laborers.

**Electronic supplementary material:**

The online version of this article (10.1186/s12992-018-0427-9) contains supplementary material, which is available to authorized users.

## Background

Human trafficking (HT), or modern slavery, is an old problem [1]. However, in the past few decades, HT became a global concern [2, 3]. The most accepted definition of HT is presented by the *Trafficking Protocol* [4]. Human trafficking was addressed by several international agreements and conferences such as the *International Agreement for the Suppression of White Slave Traffic* (1904) [5], *International Agreement for the Suppression of Traffic in Women and Children* (1927) [6], the *Trafficking Protocol* (2000), and the *Protocol against Smuggling of Migrants by Land, Sea, and Air* (2000) [4]. The last two protocols recognized HT as a transnational crime rather than just human rights or migration issue [7]. Different countries used the UN *Trafficking Protocol* to develop criminal codes for HT. As of 2016, over 150 countries had criminal laws for HT [8]. In the USA, the *Office to Monitor and Combat Trafficking in Persons* releases an annual report (TIP Report) about international efforts to combat trafficking. The European Union adopted the *Brussels Declaration on Preventing and Combating Trafficking in Human Beings* which aimed to fight HT in Europe [9]. Other non-European and non-American countries developed their own national policies and protocols to criminalize and fight HT [10].

Sex trafficking is one of the most common forms of HT with more than half a million women being trafficked every year [6]. Forced labor, child labor, child soldiers, debt bondage, involuntary domestic servitude, and organ/tissue removal for transplantation are other common forms of HT [11]. Forced prostitution represents the highest percentage of HT victims with the majority being women from Eastern Europe, Central Asia, and North and South America [8, 12, 13]. In contrast, the majority of victims of forced labor are men from Africa, the Middle East, and Asia [8, 12]. International organizations distinguish between HT and smuggling. In smuggling, the actor takes part in the process while HT involves some deception or coercion [14]. Unlike HT, migration and smuggling are less often involved with a criminal organization [14].

Human trafficking has a global dimension despite that trafficking could occur within the same country [8]. Human trafficking affects people of all races, religions, social class, and education. It often results in mental health disorders and life-threatening infections [8, 15–20]. For example, a study of 207 trafficked women from 14 countries reported that 95% of women had experienced physical and/or sexual violence [21]. A study on trafficked Nepalese girls and women found that 23% of them tested positive for HIV [22]. Despite its negative social, health, and legal consequences, HT is a growing business for criminal organizations [21, 23]. Poverty, social injustice, disasters, substance abuse, family breakdown, and homelessness are major push factors for victims [24–26]. Globalization has created a larger gap between developing and developed countries with poverty and marginalization being major push factors for victims [1].

The International Labor Organization (ILO) reported 12.3 million trafficking victims in 2005, 21 million victims in 2012, and 40.3 million victims in 2016 [27]. The 2016 report of the ILO estimated that 40.3 million people in modern slavery, including 24.9 in forced labor and 15.4 million in the forced marriage [28]. Out of the 24.9 million people trapped in forced labor, 16 million people in the private sector such as domestic work, construction or agriculture; 4.8 million persons in forced sexual exploitation, and 4 million persons in forced labor imposed by state authorities [27]. The ILO considers the Asia-Pacific region to have the largest number of victims of HT followed by Africa and Latin America [28]. It was estimated that over 40% of trafficked victims were detected in their own countries and that 71% of the trafficked victims were women while 28% were children [8].

Bibliometric analysis is a research method used to shed light on research activity [29–31]. It differs from systematic reviews, which aim to answer a specific research question based on a selected group of articles [32, 33]. It also differs from scoping reviews, which aim to identify the nature and extent of research evidence [34, 35]. Bibliometric studies were carried out to provide a snapshot of national and international contribution to literature [36] and to advance information and science [37]. Bibliometric indicators assess national and international efforts carried out to achieve a particular goal. Several bibliometric studies on migrants and refugees have been published [38–40]. However, none was carried out on HT. Therefore, the aim of this study was to analyze published literature on HT. Such a study will shed light on the global research activity and discussed themes in literature on HT. Bibliometric analysis may be useful for health authorities and UN agencies interested in mapping and identifying research gaps within the HT research landscape, which is important for advancing an evidence-informed research agenda. Findings of a bibliometric analysis are difficult to obtain by other research methods such as systematic or scoping reviews. For example, a recent systematic review of research methods on HT and health concluded that despite the presence of various quantitative and qualitative data collection and analysis methods, the implemented methodological approaches have limitations that affect what is known about HT and health [41]. Most available literature on HT is based on emotional stories rather than on empirical research [42]. Bibliometric analysis would give an accurate quantitative analysis of literature on HT that would serve as a basis for future studies. The findings of a bibliometric study on HT will shed light on the evolution, volume, and scope of research on HT and will help identify countries and regions lagging behind in this field [5]. A bibliometric analysis of literature on HT will also give detailed information on the number of publication on each type of HT and therefore a direct academic and research effort as well as funding towards less-researched types of HT.

## Methods

### Source of information

In the current study, SciVerse Scopus was selected to accomplish the objectives. Scopus is a bibliographic database created by Elsevier in November 2004 [43]. With 22,800 titles from over 5000 international publishers, Scopus delivers the most comprehensive view of the world’s research output in the fields of science, technology, medicine, social science and arts and humanities [43]. Scopus database includes all MEDLINE journals and allows for citation analysis [44, 45]. Scopus database has several operating functions that facilitate bibliometric analysis. Such operating functions include journal name, type of document, year of publication, authors and their affiliations, the number of citations, and *h*-index metrics for documents [46, 47].

### Study design

A bibliometric method was implemented. The study period was set from 2000 to 2017. The study period was determined based on the assumption that HT has attracted a lot of public attention after the introducing the UN *Trafficking Protocol* ratified in December 2000.

### Search strategy

In bibliometric analysis, selection of keywords is of utmost importance, as these keywords have a direct impact on the findings and results. In the current study, search strategy was based on title or title/abstract search with certain constraints to minimize false-positive results (Table 1**)**. Keywords were obtained from previously published literature on HT [20, 48–51]. The search strategy was limited to documents published in academic journals but was not restricted to any language. The online search was performed on June 03, 2018.

**Table 1:** Research strategy and keywords used to retrieve documents in the field of human trafficking

Search method	Keywords	Constraints	Exclusion
Title search	TITLE(“modern slavery” or “trade in human being” or “human trafficking” OR “trafficking in human” OR “lab*r trafficking” OR “forced lab*r” OR “compulsory lab*r” OR “sex* traffick*” OR “sex* trade*” OR “trafficking in persons” OR “traffick* person*” OR “traffick* wom*” OR “traffick* people” OR “traffick* girl*” OR “wom*n traffick*” OR “child* traffick*” OR “organ traffick*” OR “transplant tourism” OR “trafficking in wom*” OR “traffick* in organ*” OR “forced prostit*” OR “trafficking of child*” OR “trafficking of wom*” OR “sex* slavery” OR “commercial sexual exploitation” OR “lab*r traffick*” OR “organ trade” OR “forced marriage” OR “bonded lab*r” OR “child harvesting” OR “modern slavery” OR “child prostitut*” or “forced prostitut*” or “domestic servitude” or “debt bondage” or “debt slavery” or “bonded lab*r” or “child pornography”)	NONE	AND NOT TITLE-ABSTRACT (insect or droso* OR sexta OR cell OR molecular OR biology OR leukocyte* OR DNA)
OR			
Title search	TITLE (“tissue removal” OR “ova removal” OR slave* OR slavery OR servitude OR “child lab*r” OR “cross border traffick*” OR “child soldier*” OR anti-trafficking OR “human abduction”)	AND ALL (“human trafficking” OR “trafficking in human” OR “forced lab*r” OR “forced sex*” OR “forced prostitution” OR kidnap* or abduct* OR “child sex* or exploitation”)	AND NOT TITLE-ABSTRACT (insect or droso* OR sexta OR cell OR molecular OR biology OR leukocyte* OR DNA)
OR			
Title-Abstract search	TITLE-ABSTRACT (traffick*) AND TITLE-ABSTRACT (“in human” OR “human being*”)	AND ALL (“human trafficking” OR “trafficking in human*”)	AND NOT TITLE-ABS-KEY (phallus OR pgd OR cell OR molecule OR gene OR *cytes OR dna OR nucle* OR droso* OR sexta OR memberane OR “trade-off*” or insect) AND NOT SRCTITLE (insect OR physiology OR biology OR biochemistry OR cellular OR evolution*)
AND			
Limit	1. Source type = journal 2. *Time* interval = 2000–2017		

### Estimation of the number of health- and non-health-related documents

Using the function designated as “subject area”; it was possible to estimate the number of HT documents in health field, defined as documents in the following subject areas: medicine, nursing, psychology, pharmacology, neuroscience, general health, microbiology/immunology, biochemistry, and dentistry. The total number of health and non-health documents was greater than the total number of the retrieved documents because some journals are indexed in both medicine and social sciences; e.g. the *Journal of Social Medicine* which is categorized in both social science and medicine. Health-related documents were further divided into different domains such as mental health, health policy and systems, maternal and reproductive health, non-communicable diseases, and infectious diseases [38].

### Bibliometric indicators, analytics, and mapping

In the current study, bibliometric indicators were presented as ten most active countries, institutions, journals, authors, and ten most cited documents. The choice of number ten as a threshold to list the results was an arbitrary selection that has been used in previously published bibliometric studies [40, 52–54].

### International collaboration

Research collaboration was assessed using author affiliation. For example, documents with authors having different country affiliations represent international research collaboration while documents with authors having the same country affiliation represent intra-country collaboration. Scopus allows for segregation of documents based on authoraffiliation. Therefore, for each country, the number of documents with international authors was calculated as a percentage of the total number of documents published by that country. Documents with international authors were referred to as multiple country publications (MCP) and represented the extent of international collaboration [55–58].

### Bibliometric visualization maps

Co-authorship analysis, international collaboration, and keyword analysis were presented as network visualization maps using VOSviewer (Leiden University, Leiden, Netherlands) [59, 60].The Statistical Package for Social Sciences (SPSS) (SPSS, Chicago, Illinois) was used to generate line presentation of the annual growth of publications. ArcGIS 10.1 software (Esri; USA) is a geographic information system used in bibliometric studies to map worldwide research contribution. The GIS map allows for a better understanding of regional contribution to a specific scientific topic.

## Results

### Growth of publications and typology of documents

In total, 2044 documents were retrieved (Additional file 1). The retrieved documents were of eight different types: research articles (1553; 76.0%), reviews (280; 13.7%); notes (66; 3.2%); editorials (46; 2.3%), letters (30; 1.5%); short surveys (23; 1.1%), conference papers (22; 1.1%), and unclassified (24; 1.2%).

**Fig. 1:**
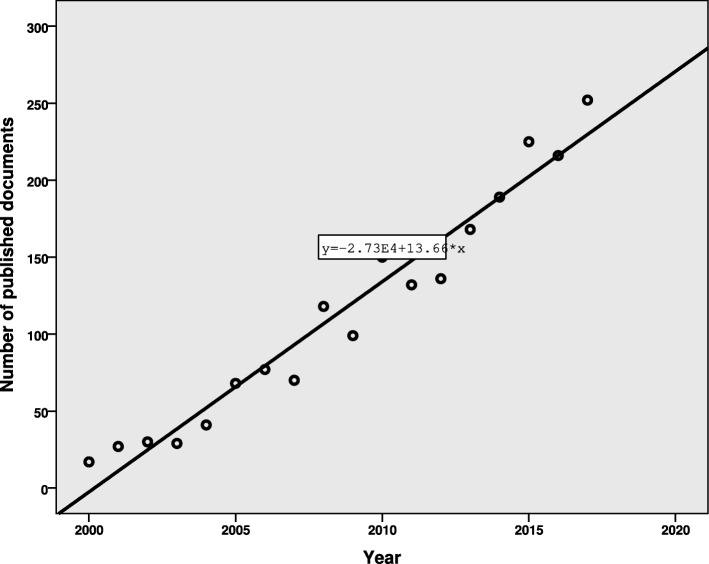
Total number of published documents from 2000 to 2017

The number of retrieved documents have significantly increased during the study period (linear regression: β = .978, *P* < .001, *R*^2^ = .956; Figure 1). If this trend continues, it was estimated that 293 documents would be published in 2020.

### Mapping the most frequent keywords and typology of trafficking

**Fig. 2:**
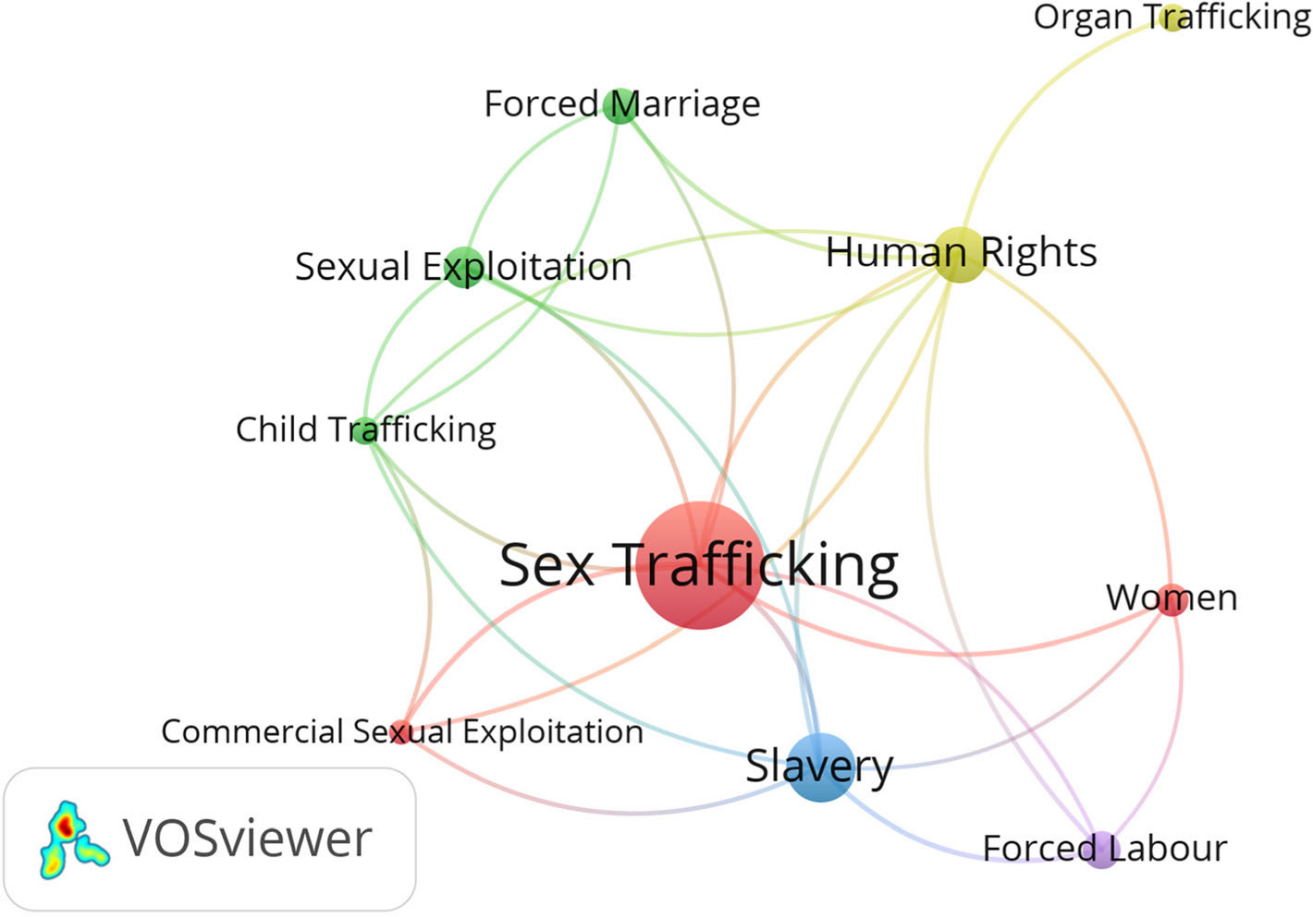
Mapping 10 most frequent keywords

Most frequent keywords were visualized (Fig. 2). The map showed five clusters: (1) organ trafficking; (2) child trafficking; (3) forced labor; (4) sex trafficking of women, and (5) slavery. Further analysis of the retrieved documents showed that 771 (37.7%) documents were about sex trafficking, 616 (30.1%) were about labor trafficking/forced labor, 199 (9.7%) were about child trafficking, and 138 (6.8%) were about organ trafficking. The remaining documents were general documents or ones that discussed multiple types of trafficking.

### Research domains

**Fig. 3:**
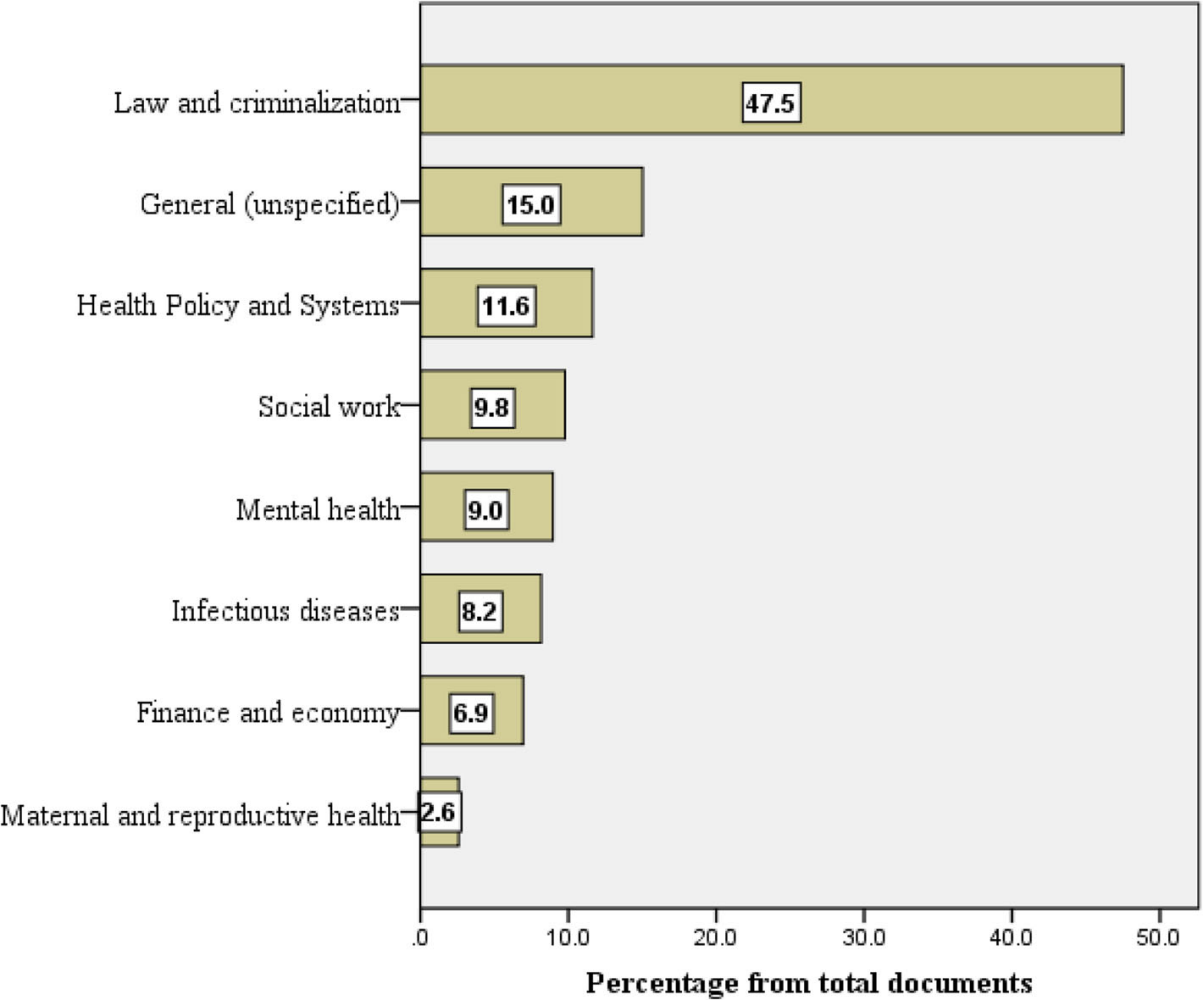
Research domains in the retrieved literature

In total, 707 (34.6%) documents were in the health field while 1526 (74.7%) were in social sciences and humanities taking into consideration certain limited overlap between the health and health research domains. Analysis of the retrieved showed that 971 (47.5%) documents were about law and criminology, 238 (11.6%) were about health policy and systems, 200 (9.8%) were about social work, and 183 (9.0%) were about mental health (Fig. 3**).**

### Top 10 active countries

**Fig. 4:**
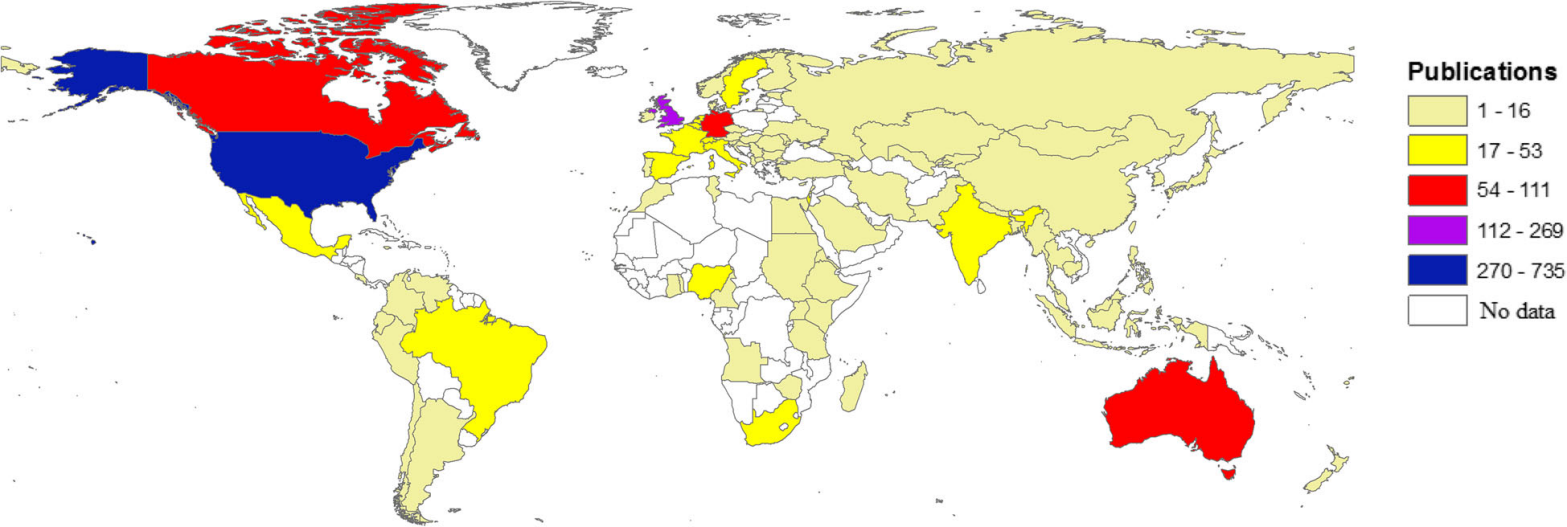
Geographic distribution of publications based on the country affiliation of authors. The following is the color-coding for the map

Authors from 91 different countries participated in publishing the retrieved documents. The top 10 active countries participated in publishing 1478 (72.3%) documents (Table 2). The United States ranked first (735; 36.0%) regarding the number of published documents. Five countries in the top 10 active list were in Western Europe, two were in Northern America, one in the Western Pacific region, one in Southeast Asia, and one in Latin America. The geographic distribution of the retrieved document showed that world regions with a high prevalence of HT, such as South East Asia, East Europe, Africa, and Latin America [8], had the least research contribution (Fig. 4).

**Table 2:** Top 10 active countries and international research collaboration

Rank	Country	Frequency (%) *N* = 2044	Number of collaborating countries	SCP	MCP
1st	United States	735 (36.0)	43	644 (87.6)	91 (12.4)
2nd	United Kingdom	269 (13.2)	28	222 (82.5)	47 (17.5)
3rd	Canada	111 (5.4)	20	88 (79.3)	23 (20.7)
4th	Australia	84 (4.1)	19	68 (81.0)	16 (19.0)
5th	Germany	75 (3.7)	13	60 (80.0)	15 (20.0)
6th	Netherlands	53 (2.6)	10	45 (84.9)	8 (15.1)
7th	Brazil	43 (2.1)	10	38 (88.4)	5 (11.6)
8th	Italy	38 (1.9)	9	31 (81.6)	7 (18.4)
9th	India	37 (1.8)	15	19 (51.4)	18 (48.6)
10th	France	33 (1.6)	7	25 (75.8)	8 (24.2)
		1478 (72.3)		1240 (83.9)	238 (16.1)

*SCP* single country collaboration = intra-country collaboration, *MCP* multiple country collaboration = international collaboration

### International collaboration

Analysis of international research collaboration for the top 10 active countries showed that India had the highest percentage of documents (49%) with international research collaboration. In contrast, Brazil had the least percentage of documents (12%) with international research collaboration. For the US, which led in the number of publications, only 12.5% of documents with US authors included authors from other countries. Of the 1478 publications produced by the top 10 active countries, there were only 238 (16.1%) publications with international collaboration.

### Top 10 active institutions

*Harvard University* (USA) (*n* = 39; 1.9%) was the most active institution in this field followed by *University of British Columbia* (Canada) and *London School of Hygiene & Tropical Medicine* (UK) (Table 3). The top 10 active list of institutions included four in the USA, three in Canada, and three in the UK.

**Table 3:** Top 10 active institutions in the field of human trafficking

Rank	Institution	Frequency (%) N = 2044	Country
1st	Harvard Medical School	39 (1.9)	USA
2nd	The University of British Columbia	26 (1.3)	Canada
3rd	London School of Hygiene & Tropical Medicine	22 (1.1)	UK
4th	Massachusetts General Hospital	20 (1.0)	USA
5th	King’s College London	18 (0.9)	UK
5th	University of Oxford	18 (0.9)	UK
7th	Johns Hopkins University	15 (0.7)	USA
8th	University of Toronto	14 (0.7)	Canada
9th	George Mason University	13 (0.6)	USA
9th	York University	13 (0.6)	Canada

### Authorship analysis

In total, 3920 authors participated in publishing the retrieved documents, giving an average of 1.9 authors per document. Approximately 61% (*n* = 1250) documents were single-authored publications, 20.0% (*n* = 408) were two-authored publications, while the remaining 18.9% (*n* = 386) were multi-authored publications (≥ 3 authors per document). Professor Cathy Zimmerman (UK) was the most active author (22; 1.1%). Professor Jay Silverman (USA) ranked second (17; 0.8%) (Table 4).

**Table 4:** Top 10 active authors in the field of human trafficking

Rank^a^	Author	Frequency (%) N = 2044	Affiliation
1st	Zimmerman, C.	22 (1.1)	London School of Hygiene & Tropical Medicine, Department of Global Health and Development, London, United Kingdom
2nd	Silverman, J.G.	17 (0.8)	Center on Gender Equity and Health, Division of Global Public Health. University of California, San Diego, San Diego, United States
3rd	Oram, S.	16 (0.8)	Health Service and Population Research, King’s College London, London, United Kingdom
4th	Decker, M.R.	14 (0.7)	Johns Hopkins Bloomberg School of Public Health, Department of Population, Baltimore, United States
5th	Reid, J.A.	13 (0.7)	University of South Florida St. Petersburg, St Petersburg, United States
6th	Raj, A.	12 (0.6)	University of California, San Diego, Department of Medicine, San Diego, United States
7th	Delmonico, F.L.	11 (0.5)	Organisation Mondiale de la Sante, New England Donor Services, Geneve, Switzerland
8th	Howard, L.M.	9 (0.4)	King’s College London, London, United Kingdom
9th	Cho, S.Y.	8 (0.4)	Universitat Marburg, Marburg, Germany
9th	Gupta, J.	8 (0.4)	George Mason University, Department of Global and Community Health, Fairfax, United States

^a^Authors with equal research output were given the same rank, and then a gap is left in the ranking numbers

### Research networks

**Fig. 5:**
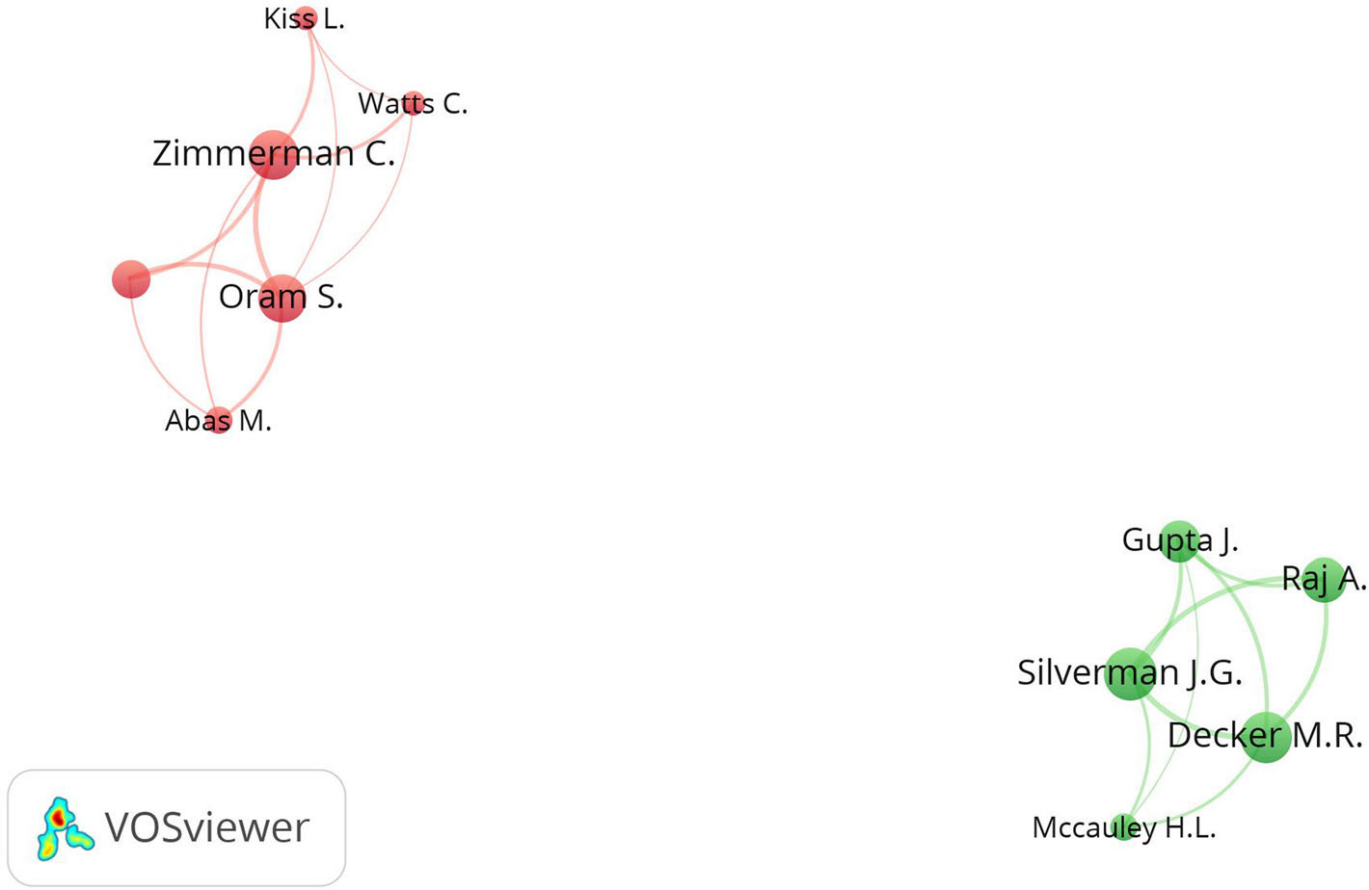
Networks of active authors who published at least five publications and exist in a research network with a minimum of five authors

Mapping research networks for authors with a minimum research output of five documents showed only two research clusters (Fig. 5). The first cluster (red) included six authors with Zimmerman, C. as a member in this cluster. The second cluster (green) included five authors with Silverman, J as a member of this cluster.

### Preferred journals for publishing documents about HT

The retrieved documents were published in 998 different journals. The *International Migration* was the most active journal in this field. Top 10 active journals belonged to different fields including migration, criminology, medicine, social studies, gynecology, transplantation, violence, and human rights (Table 5). The majority of active journals were based in the USA. Documents published in *Transplantation* (25.5) journal received the highest number of citations per article followed by those published in *International Migration* (22.5).

**Table 5:** Top 10 active journals in the field of human trafficking

Rank^a^	Journal	Frequency (%) N = 2044	C/A^b^		Subject area (category)^c^
1st	International Migration	35 (1.7)	22.5	UK	Social sciences (demography)
2nd	Trends In Organized Crime	25 (1.2)	7.6	USA	Social sciences (Law)
3rd	Crime Law And Social Change	24 (1.2)	7.3	Netherlands	Medicine (Pathology and Forensic Medicine)
4th	Social Inclusion	20 (1.0)	1.7	Portugal	Psychology (social psychology)
5th	Lancet	19 (0.9)	6.3	UK	Medicine
6th	Cadernos Pagu	15 (0.7)	3.1	Brazil	Social Sciences (Gender studies)
6th	International Social Work	15 (0.7)	12.6	USA	Social Sciences (social and political sciences)
8th	Human Rights Review	13 (0.6)	7.3	Netherlands	Social sciences (Law)
8th	Osteuropa	13 (0.6)	1.2	Germany	(social and political sciences)
10th	European Journal Of Criminology	12 (0.6)	9.8	USA	Social sciences (Law)
10th	European Journal On Criminal Policy And Research	12 (0.6)	12.3	Netherlands	Social sciences (Law)
10th	International Journal Of Gynecology And Obstetrics	12 (0.6)	17.8	UK	Medicine (Gynecology And Obstetrics)
10th	Journal Of International Criminal Justice	12 (0.6)	6.2	UK	Social sciences (Law)
10th	Journal Of Interpersonal Violence	12 (0.6)	10.9	USA	Psychology (clinical psychology)
10th	Transplantation	12 (0.6)	25.8	USA	Medicine (Transplantation)
10th	Women And Criminal Justice	12 (0.6)	7.5	USA	Social sciences (Law and Gender studies)

^a^ Journals with equal research output were given the same rank, and then a gap is left in the ranking numbers

^b^Number of citations per article

^c^Obtained from Scimago Journal and Country Rank

### Citation analysis

The retrieved documents received 15,505 citations giving a mean of 7.6 citations per document. The *h*-index of the retrieved documents was 48. The document that received the highest number of citations was published in 2007 in *Politics and Society*. Top ten cited documents discussed topics about organ trafficking, mental health, and HIV infections of HT victims. Table 6 showed the list of top 10 cited documents [22, 61–70].

**Table 6:** Top 10 cited documents in the field of human trafficking

Ranka^a^	Title	Source title	Number of citations	Document Type
1st	The social construction of sex trafficking: Ideology and institutionalization of a moral crusade	Politics and Society	246	Article
2nd	The state of the international organ trade: A provisional picture based on integration of available information	Bulletin of the World Health Organization	172	Review
3rd	Organ trafficking and transplant tourism: A commentary on the global realities	American Journal of Transplantation	146	Note
4th	The Swedish law that prohibits the purchase of sexual services: Best practices for prevention of prostitution and trafficking in human beings	Violence Against Women	132	Review
5th	The ‘butner study’ redux: A report of the incidence of hands-on child victimization by child pornography offenders	Journal of Family Violence	123	Article
5th	Will the real sex slave please stand up?	Feminist Review	123	Article
7th	(Un)popular strangers and crises (un)bounded: Discourses of sex-trafficking, the European political community and the panicked state of the modern state	European Journal of International Relations	116	Review
8th	HIV prevalence and predictors of infection in sex-trafficked nepalese girls and women	Journal of the American Medical Association	113	Article
8th	The perverse politics of four-letter words: Risk and pity in the securitisation of human trafficking	Millennium: Journal of International Studies	113	Article
10th	Prevalence and risk of violence and the physical, mental, and sexual health problems associated with human trafficking: Systematic review	PLoS Medicine	102	Article
10th	Transplant tourism: Outcomes of United States residents who undergo kidney transplantation overseas	Transplantation	102	Conference Paper

^a^Documents with equal number of citations were given the same rank, and then a gap is left in the ranking numbers

## Discussion

### Volume and growth of publications

The current study aimed to assess and analyze published literature on HT. Quantitative analysis of literature on HT is complicated by the lack of academic and legal consensus on the definition of HT as well as the unclear distinction between trafficked victims, exploited people, and vulnerable migrants [71]. For example, some researchers consider illegal migrants who end up in prostitution as victims of HT [72, 73]. Despite these technical difficulties, the current study was the first to analyze volume, growth, research trends, and research domains of literature on HT published in academic journals.

The findings of the current study showed that the total number of retrieved documents on HT was low when compared to the size of literature on the 21 million people described as refugees [74]. The number of publications on HT could be attributed to the methodological, operational, criminal, and hidden nature of HT [20]. The limited research collaboration, as evident from the mean number of authors per document and the percentage of documents with international authors, is another potential reason for the limited number of publications on HT. The finding regarding international research collaboration is difficult to explain but could be attributed to lack of communications, motivation, funding, or lack of international conferences that could help gather experts in this field [75, 76].

### Health versus non-health related research

The current study showed that there was an underrepresentation of health-related research on HT despite the potential threat of HT to national health security [1]. Health is a subject that has been neglected in anti-trafficking work efforts compared to activities in the fields of immigration and law enforcement [77, 78]. Many experts in health and HT agree that the health sector has had limited engagement in trafficking dialogues and research [78] despite that HT is being considered as a threat to global health security [79]. The presence of sexually transmitted diseases and tuberculosis among trafficked victims place the general population at risk [80, 81].

The findings of the current study regarding the under-representation of labor trafficking relative to sex trafficking were in agreement several previously published studies [18, 82]. Promotion and advancement of research on health aspects of HT require intensive involvement of public health experts in the global debate about HT. Public health experts and editors of medical and health journals need to create an international forum to encourage researchers from different parts of the world to get involved in health research about HT. This will benefit global public health agendas by shedding light on aspects related to HT such as labor exploitation and smuggling in Africa, Asia, and the Middle East where labor exploitation might not be considered illegal or a human right violation [83]. Establishing research networks that include scholars from destination and source countries will create a more detailed analysis of health aspects of HT.

### The source versus destination countries

The current study showed that the bulk of the retrieved literature and the topics discussed represent the perspective of the destination rather than source countries. It is believed that the source countries are ones with no or minimum level of democracy, high levels of corruption, and poor economic growth, which make engagement in research and academic investigation an unaffordable luxury. The source countries might not have enough public health researchers or experts HT, which influence their contribution to literature in this field. It is recommended that the source countries place migration and trafficking in their national and foreign policy agendas. Healthcare professionals, social care specialists, and experts in global health diplomacy in source countries need to get engaged in developing evidence-based information about methods used by traffickers in recruiting their victims from source countries [84]. Legal and labor experts need to provide detailed information for migrants to increase their awareness of labor exploitation and trafficking [85]. Awareness of being a potential victim to traffickers is important in source countries, particularly those with a humanitarian crisis or armed conflict where young desperate men and women will take a quick decision regarding migration in hope of a better life and better wages [86]. For destination countries, healthcare professionals, and public health experts need to identify working conditions and occupational health hazards of migrant workers who might end up being victims of HT. Policymakers need to develop strict regulation to provide migrants with legal and health protection equivalent to that of domestic workers [87]. Health policymakers need to develop mechanisms to discover victims of HT once they attend a healthcare facility [88] and offer them appropriate health services and social as well as financial support and compensation. Experts in criminology need to develop detailed criminal codes based on international diplomatic cooperation to fight organizations involved in HT [89, 90].

### Types of trafficking

In the current study, published literature on sex trafficking dominated the field of HT. The over-representation of sex trafficking suggests that research related to other forms of trafficking, particularly labor trafficking is being under-researched. Trafficked laborers live and work in harsh and mostly unhealthy conditions that might involve confiscation of passports, low wages, sex slavery, and deprivation of basic health need [91–94]. Research domain in labor trafficking needs to be strengthened, supported, funded, and encouraged to generate more evidence-based data in this field.

### Study limitations

The current study has a few limitations. Using Scopus database to retrieve literature on HT created a certain bias toward countries with a large number of journals indexed in Scopus. Scopus is biased toward academic journals in which documents are published in English. Therefore, documents in HT published in non-English were not retrieved. The current study, like all other bibliometric studies, did not include grey literature. The search strategy used in this study might have led to some false-negative or false-positive results. Therefore, interpretation of the results should take into consideration these limitations.

## Conclusion

The current study is the first to assess research activity in the field of HT. The current study showed that health aspects of HT were under-represented compared to criminal, legal, and social aspects of HT. Similarly, research on labor trafficking was under-represented compared to sex trafficking and exploitation. The literature on HT represents the agendas of destination countries with limited contribution of countries in Asia, Latin America, Africa, the Middle East, and Eastern European. Future research plans should shed light on trafficking for non-sexual purposes. Furthermore, research networks need to be strengthened by including scholar from source and destination countries to investigate health conditions of trafficking survivors or exploited migrants. Greater investments in international research collaborations and research networks should be encouraged to help prioritize research on HT in various world regions.

## Additional file

Additional file 1: Scheme of the sequence of data extraction and analysis. Number of documents retrieved using Scopus at each step in the search strategy used to obtain the required data. (DOCX 30 kb)

## Abbreviations

HT: human trafficking
MCP: multiple country collaboration = international collaboration
SCP: single country collaboration = intra-country collaboration

## References

[1] Shelley L. Human trafficking: a global perspective. New York: Cambridge University press; 2010.

[2] Davy D (2016). Anti–human trafficking interventions: how do we know if they are working?. Am J Eval.

[3] Gulati GJ (2011). News frames and story triggers in the Media’s coverage of human trafficking. Hum Rights Rev.

[4] United Nations (UN) (2000). Protocol to Prevent, Suppress and Punish Trafficking in Persons, Especially Women and Children.

[5] Kangaspunta K. Was trafficking in persons really criminalised? Anti-Trafficking Rev. 2015;4.

[6] Whitman A, Gray DH. Transnational Human Trafficking. Global Secur Stud. 2015;6(3).

[7] Shelley L. 6 human trafficking as a form of transnational crime. In: Human Trafficking. Portland: Willan; 2013. p. 128–49.

[8] United Nations Office on Drugs and Crime (UNODC): Global Report in Trafficking in Persons 2016. 2016.

[9] Laczko F (2005). Data and research on human trafficking. Int Migr.

[10] Amahazion F (2015). Human trafficking: the need for human rights and government effectiveness in enforcing anti-trafficking. Global crime.

[11] Zimmerman C, Kiss L (2017). Human trafficking and exploitation: a Global Health concern. PLoS Med.

[12] Honeyman KL, Stukas AA, Marques MD (2016). Human trafficking: factors that influence willingness to combat the issue. J Appl Soc Psychol.

[13] Tripp TM, McMahon-Howard J (2016). Perception vs. reality: the relationship between organized crime and human trafficking in metropolitan Atlanta. Am J Crim Justice.

[14] Aronowitz AA (2001). Smuggling and trafficking in human beings: the phenomenon, the markets that drive it and the Organisations that promote it. European J Crim Policy Res.

[15] Ottisova L, Smith P, Shetty H, Stahl D, Downs J, Oram S (2018). Psychological consequences of child trafficking: an historical cohort study of trafficked children in contact with secondary mental health services. PLoS One.

[16] Charteris A, German C, Hansrani Z, Li E, Merchant J (2018). Health at Risk: Health Implications of Human Trafficking in the Context of Globalization and Migration.

[17] Henry AML, Grodin MA (2018). Human trafficking: a health and human rights agenda. Ann Intern Med.

[18] Cockbain E, Bowers K, Dimitrova G. Human trafficking for labour exploitation: the results of a two-phase systematic review mapping the European evidence base and Synthesising key scientific research evidence. J Exp Criminol. 2018:1–42. 10.1007/s11292-017-9321-3 PMC6417370 30956630

[19] Hemmings S, Jakobowitz S, Abas M, Bick D, Howard LM, Stanley N, Zimmerman C, Oram S. Responding to the Health Needs of Survivors of Human Trafficking: A Systematic Review. BMC Health Serv Res. 2016;16(1). 10.1186/s12913-016-1538-8 PMC4966814 27473258

[20] Ottisova L, Hemmings S, Howard LM, Zimmerman C, Oram S (2016). Prevalence and risk of violence and the mental, physical and sexual health problems associated with human trafficking: an updated systematic review. Epidemiol Psychiatr Sci.

[21] Zimmerman C, Hossain M, Yun K, Roche B, Morison L, Watts C. Stolen smiles: a summary report on the physical and psychological health consequences of women and adolescents trafficked in Europe. Stolen smiles. 2006.

[22] Silverman JG, Decker MR, Gupta J, Maheshwari A, Willis BM, Raj A (2007). Hiv prevalence and predictors of infection in sex-trafficked Nepalese girls and women. Jama.

[23] Wheaton EM, Schauer EJ, Galli TV (2010). Economics of human trafficking. Int Migr.

[24] Cho S-Y (2015). Modelling for determinants of human trafficking.

[25] Okech D, McGarity SV, Hansen N, Burns AC, Howard W (2018). Financial capability and sociodemographic factors among survivors of human trafficking. J Evid informed Soc Work.

[26] Wolfe D, Greeson J, Wasch S, Treglia D (2018). Human trafficking prevalence and child welfare risk factors among homeless youth a Multi-City study.

[27] International Labour Organization (ILO); Forced Labour, Modern Slavery and Human Trafficking 2016 [Cited 2018 June 02, 2018].

[28] International Labour Organization (ILO) (2017). Global Estimates of Modern Slavery: Forced Labour and Forced Marriage.

[29] Sweileh WM, Al-Jabi SW, AbuTaha AS, Zyoud SH, Anayah FMA, Sawalha AF (2017). Bibliometric analysis of worldwide scientific literature in Mobile - health: 2006-2016. BMC Med Inform Decis Mak.

[30] Sweileh WM, AbuTaha AS, Sawalha AF, Al-Khalil S, Al-Jabi SW, Zyoud SH. Bibliometric Analysis of Worldwide Publications on Multi-, Extensively, and Totally Drug - Resistant Tuberculosis (2006-2015). Multidiscip Respir Med. 2016, 11:45. 10.1186/s40248-016-0081-0 PMC5225617 28096979

[31] Sweileh WM, Sawalha AF, Al-Jabi SW, Zyoud SH, Shraim NY, Abu-Taha AS (2016). A bibliometric analysis of literature on malaria vector resistance: (1996 - 2015). Glob Health.

[32] Peters MD, Godfrey CM, Khalil H, McInerney P, Parker D, Soares CB (2015). Guidance for conducting systematic scoping reviews. Int J Evid Based Healthc.

[33] Møller A, Myles P (2016). What makes a good systematic review and meta-analysis?. Br J Anaesth.

[34] Grant MJ, Booth A (2009). A typology of reviews: an analysis of 14 review types and associated methodologies. Health Inf Libr J.

[35] Levac D, Colquhoun H, O'Brien KK (2010). Scoping studies: advancing the methodology. Implement Sci.

[36] Rosas SR, Kagan JM, Schouten JT, Slack PA, Trochim WM (2011). Evaluating research and impact: a bibliometric analysis of research by the Nih/Niaid Hiv/aids clinical trials networks. PLoS One.

[37] Koskinen J, Isohanni M, Paajala H, Jaaskelainen E, Nieminen P, Koponen H, Tienari P, Miettunen J (2008). How to use bibliometric methods in evaluation of scientific research? An example from Finnish schizophrenia research. Nord J Psychiatry.

[38] Sweileh WM, Wickramage K, Pottie K, Hui C, Roberts B, Sawalha AF, Zyoud SH (2018). Bibliometric analysis of global migration Health Research in peer-reviewed literature (2000-2016). BMC Public Health.

[39] Sweileh WM. Global research output on HIV/AIDS-related medication adherence from 1980 to 2017. BMC Health Serv Res. 2018;18(1):765. 10.1186/s12913-018-3568-x. 10.1186/s12913-018-3568-x PMC6180611 30305093

[40] Sweileh WM (2018). Global research output in the health of international Arab migrants (1988-2017). BMC Public Health.

[41] Cannon AC, Arcara J, Graham LM, Macy RJ (2018). Trafficking and health: a systematic review of research methods. Trauma Violence Abuse.

[42] Goździak EM. Data Matters: Issues and Challenges for Research on Traffi Cking. In: Global Human Trafficking: Routledge; 2014. p. 37–52.

[43] Elsevier; Scopus [https://www.elsevier.com/solutions/scopus/content]. Accessed 1 Sept 2018.

[44] Falagas ME, Pitsouni EI, Malietzis GA, Pappas G (2008). Comparison of Pubmed, Scopus, web of science, and Google scholar: strengths and weaknesses. FASEB J.

[45] Kulkarni AV, Aziz B, Shams I, Busse JW (2009). Comparisons of citations in web of science, Scopus, and Google scholar for articles published in general medical journals. JAMA.

[46] Hirsch JE (2005). An index to quantify an Individual's scientific research output. Proc Natl Acad Sci U S A.

[47] Agarwal A, Durairajanayagam D, Tatagari S, Esteves SC, Harlev A, Henkel R, Roychoudhury S, Homa S, Puchalt NG, Ramasamy R (2016). Bibliometrics: tracking research impact by selecting the appropriate metrics. Asian J Androl.

[48] Babik JM, Chin-Hong P (2015). Transplant Tourism: Understanding the Risks. Curr Infect Dis Rep.

[49] Coverdale J, Beresin EV, Louie AK, Balon R, Roberts LW (2016). Human trafficking and psychiatric education: a call to action. Acad Psychiatry.

[50] Muraya DN, Fry D (2015). Aftercare Services for Child Victims of sex trafficking: a systematic review of policy and practice. Trauma Violence Abuse.

[51] Varma S, Gillespie S, McCracken C, Greenbaum VJ (2015). Characteristics of child commercial sexual exploitation and sex trafficking victims presenting for medical Care in the United States. Child Abuse Negl.

[52] Sweileh WM (2017). Global research trends of World Health Organization's top eight emerging pathogens. Glob Health.

[53] Sweileh WM. Bibliometric analysis of literature in aids-related stigma and discrimination. Transl Behav Med. 2018. 10.1093/tbm/iby072 29982818

[54] Sweileh WM (2018). Bibliometric analysis of peer-reviewed literature in transgender health (1900 - 2017). BMC Int Health Hum Rights.

[55] Sweileh WM, Al-Jabi SW, Sawalha AF (2017). Bibliometric Analysis of Worldwide Publications on Antimalarial Drug Resistance (2006–2015).

[56] Sweileh WM, Al-Jabi SW, Zyoud SH, Sawalha AF (2018). Outdoor air pollution and respiratory health: a bibliometric analysis of publications in peer-reviewed journals (1900 - 2017). Multidiscip Respir Med.

[57] Zyoud SH, Sweileh WM, Awang R, Al-Jabi SW (2018). Global trends in research related to social Media in Psychology: mapping and bibliometric analysis. Int J Ment Health Syst.

[58] Zyoud SH, Waring WS, Al-Jabi SW, Sweileh WM (2017). Global Cocaine Intoxication Research Trends During 1975-2015**:** A bibliometric analysis of web of Science Publications. Subst Abuse.

[59] van Eck NJ, Waltman L (2010). Software survey: Vosviewer, a computer program for bibliometric mapping. Scientometrics.

[60] van Eck NJ, Waltman L (2011). Vosviewer Manual.

[61] Aradau C (2004). The perverse politics of four-letter words: risk and pity in the securitisation of human trafficking. Millennium.

[62] Berman J (2003). (Un)Popular Strangers and Crises (Un)Bounded: Discourses of Sex-Trafficking, the European Political Community and the Panicked State of the Modern State. Eur J Int Rel.

[63] Bourke ML, Hernandez AE (2009). The ‘Butner Study’ redux: a report of the incidence of hands-on child victimization by child pornography offenders. J Fam Violence.

[64] Budiani-Saberi DA, Delmonico FL (2008). Organ trafficking and transplant tourism: a commentary on the global realities. Am J Transplant.

[65] Canales MT, Kasiske BL, Rosenberg ME (2006). Transplant tourism: outcomes of United States residents who undergo kidney transplantation overseas. Transplantation.

[66] Ekberg G (2004). The Swedish law that prohibits the purchase of sexual services: best practices for prevention of prostitution and trafficking in human beings. Violence Against Women.

[67] O'Connell Davidson J (2006). Will the real sex slave please stand up?. Fem Rev.

[68] Oram S, Stöckl H, Busza J, Howard LM, Zimmerman C (2012). Prevalence and Risk of Violence and the Physical, Mental, and Sexual Health Problems Associated with Human Trafficking: Systematic Review. PLoS Med.

[69] Shimazono Y (2007). The state of the international organ trade: a provisional picture based on integration of available information. Bull World Health Organ.

[70] Weitzer R (2007). The social construction of sex trafficking: ideology and institutionalization of a moral crusade. Polit Soc.

[71] Oram S, Zimmerman C, Adams B, Busza J (2011). International Law, National Policymaking, and the Health of Trafficked People in the Uk. Health Human Rights.

[72] Kara S. Sex trafficking: inside the business of modern slavery: Columbia University press; 2009.

[73] Yen I (2007). Of vice and men: a new approach to eradicating sex trafficking by reducing male demand through educational programs and abolitionist legislation. J Crim L Criminol.

[74] Sweileh WM (2017). Bibliometric analysis of medicine–related publications on refugees, asylum-seekers, and internally displaced people: 2000–2015. BMC Int Health Hum Rights.

[75] Gazni A, Sugimoto CR, Didegah F (2012). Mapping world scientific collaboration: authors, institutions, and countries. J Am Soc Inf Sci Technol.

[76] Wilsdon J. Knowledge, networks and nations: global scientific collaboration in the 21st century: The Royal Society; 2011.

[77] Morrison J, Director E, Crosland B (2000). The trafficking and smuggling of refugees: the end game in European asylum policy?.

[78] Zimmerman C, Hossain M, Watts C (2011). Human trafficking and health: a conceptual model to inform policy, Intervention and Research. Soc Sci Med.

[79] Moreno MA. Human trafficking: from a Global Health perspective: San Diego State University; 2015.

[80] Tiittala P, Ristola M, Liitsola K, Ollgren J, Koponen P, Surcel HM, Hiltunen-Back E, Davidkin I, Kivelä P. Missed hepatitis b/c or syphilis diagnosis among Kurdish, Russian, and Somali origin migrants in Finland: linking a population-based survey to the national infectious disease register. BMC Infect Dis. 2018;18(1):137. 10.1186/s12879-018-3041-9 PMC5859750 29558910

[81] Decker M. R., McCauley H. L., Phuengsamran D., Janyam S., Silverman J. G. (2010). Sex trafficking, sexual risk, sexually transmitted infection and reproductive health among female sex workers in Thailand. Journal of Epidemiology & Community Health.

[82] Surtees R. Trafficking in men–cases from Belarus and Ukraine. IOM Migr Res Ser. 2008:17–9.

[83] Kharel U. The global epidemic of occupational injuries: counts, costs, and compensation: The Pardee RAND Graduate School; 2016.

[84] Novotny TE, Kickbusch I. 21st Century Global Health Diplomacy, vol. 3: World Scientific; 2013.

[85] Azad A. Recruitment of migrant Workers in Bangladesh: elements of human trafficking for labor exploitation. J Hum Trafficking. 2018:1–21.

[86] International Labour Organization (ILO) (2015). Addressing Human Trafficking and Exploitation in Times of Crisis- Evidence and Recommendations for Further Action to Protect Vulnerable and Mobile Populations I December 2015.

[87] Marks E, Olsen A. Policy and practice: the role of trade unions in reducing migrant workers’ vulnerability to forced labour and human trafficking in the greater Mekong subregion. Anti-Trafficking Rev. 2015;5.

[88] Chang KS, Hayashi AS: The Role of Community Health Centers in Addressing Human Trafficking. In: Human Trafficking Is a Public Health Issue*.* edn.: Springer; 2017: 347–362.

[89] van der Leun J, Bedrijfsvoering S (2017). Criminal policy, migration policy and human trafficking. Addressing the Dark Figure.

[90] Stolz BA. Criminal justice policy and transnational crime: the case of anti–human trafficking policy. US Criminal Justice Policy. 2015;337.

[91] Barrick K, Lattimore PK, Pitts WJ, Zhang SX (2014). When farmworkers and advocates see trafficking but law enforcement does not: challenges in identifying labor trafficking in North Carolina. Crime Law Soc Chang.

[92] Bélanger D (2014). Labor migration and trafficking among Vietnamese migrants in Asia. Ann Am Acad Pol Soc Sci.

[93] Brennan D (2014). Trafficking, scandal, and abuse of migrant Workers in Argentina and the United States. ANNALS Am Acad Political Soc Sci.

[94] Joarder MAM, Miller PW (2014). The experiences of migrants trafficked from Bangladesh. Ann Am Acad Political Soc Sci.

