# Supplementary material for: Research trends on human trafficking: a bibliometric analysis using Scopus database
Source: Global Health. 2018 Nov 8;14:106. doi: 10.1186/s12992-018-0427-9 (PMC6225706; doi:10.1186/s12992-018-0427-9)
Supplement: Supplementary file 1 — Scheme of the sequence of data extraction and analysis. Number of documents retrieved using Scopus at each step in the search strategy used to obtain the required data. (DOCX 30 kb) [file 12992_2018_427_MOESM1_ESM.docx]

**Additional file**

**(Additional 1)**

**Scheme of the sequence of data extraction and analysis**

**Exclude false positive keywords listed in Table 1**

**N = 3068**

**Limit to documents published in academic journals**

**N = 2311**

**Search strategy using the keywords listed in Table 1**

**N = 3178**

**Search strategy using the keywords listed in Table 1**

**N = 3178**

**Search strategy using the keywords listed in Table 1**

**N = 3178**

1. **Limit to health-related subject areas**

**N = 707**

1. **Limit to non-health-related subject areas**

**N = 1526**

**Research domains of health-related documents**

Health policy and systems (N = 238); Mental health (N = 183); Infectious diseases (N = 167); Maternal and reproductive health (N = 53)

**Research domains of non-health-related documents**

Law and criminalization (N=971); Social work (N=200)

**General;** N = 306 [includes unspecified health (N=117) and non-health documents (N=189)]

**Limit to study period from 2000 to 2017**

**N = 2044**
